# Annual Trends in the Incidence and Prevalence of Alzheimer's Disease in South Korea: A Nationwide Cohort Study

**DOI:** 10.3389/fneur.2022.883549

**Published:** 2022-05-19

**Authors:** Min Seok Baek, Han-Kyeol Kim, Kyungdo Han, Hyuk-Sung Kwon, Han Kyu Na, Chul Hyoung Lyoo, Hanna Cho

**Affiliations:** ^1^Department of Neurology, Wonju Severance Christian Hospital, Yonsei University Wonju College of Medicine, Wonju, South Korea; ^2^Department of Neurology, Gangnam Severance Hospital, Yonsei University College of Medicine, Seoul, South Korea; ^3^Department of Statistics and Actuarial Science, Soongsil University, Seoul, South Korea

**Keywords:** Alzheimer's disease, prevalence, incidence, risk factors, trends, South Korea

## Abstract

Despite recent studies suggesting a declining incidence and prevalence of dementia on a global scale, epidemiologic results with respect to Alzheimer's disease (AD) are lacking due to the methodological limitations inherent to conducting large-scale cohort investigations of this topic. The aim of the current study was to investigate the incidence and prevalence of AD in Korea. We conducted a secondary analysis within the National Health Insurance System (NHIS) database, a unique resource that reports medical information for the entire Korean population. AD diagnoses as well as evaluations of vascular risks were defined based on International Statistical Classification of Diseases (ICD-10) codes along with prescription records. The cut-off age for diagnosing AD was defined as the age of the patient's highest Youden index. In this study, the incidence and prevalence of AD in the Korean population aged 40 years or older showed an overall increase between 2006 and 2015. Although both older and younger age groups showed an increase in the incidence and prevalence of AD, the highest increase was observed in older age groups. Based on the highest Youden's index value (sensitivity + specificity – 1), the cut-off value for the diagnosis of AD was 69 years with an area under the curve (AUC) of 0.92. We found that the incidence of AD was higher in individuals with underlying vascular risks. However, in recent years, the prevalence of AD was conversely found to be lower in individuals with hypertension or dyslipidemia. Despite efforts toward reducing the number of AD cases through educational, policy, and various public health and preventive medicine interventions, the incidence and prevalence of AD continues to grow in Korea. Efforts aimed at early diagnosis and the modification of underlying risks may be critical to reducing the socioeconomic burden of AD.

## Introduction

Dementia represents a common and diverse set of neurological conditions and presents a critically important concern from a public health, economic, social, and political perspective. Currently, dementia affects 50 million people worldwide. This (1, 2) number is projected to grow to 82 million by 2030 and to 152 million by 2050 due to the global aging of the population (3). As part of an effort to reduce the associated socio-economic burden, governments worldwide have enacted policies aimed at the assessment, prevention, and treatment of dementia. Previous epidemiological studies evaluating the incidence and prevalence of dementia have demonstrated conflicting results. Some studies conducted in Europe and the United States have suggested a decreasing trend in both the incidence and prevalence of dementia (4–7), whereas other studies conducted in these countries and elsewhere shown conflicting findings (8–10). Changes in dementia incidence and prevalence trends may be attributed to various interventions, including improved education as well as more efficacious treatment for vascular risk factors (4, 5, 7). However, study results based on all-cause dementia diagnoses present inherent difficulties in the interpretation of results as there are many different types of dementia, all of which may present with different responses or modifying factors upon risk management. Moreover, the relative proportions of different types of dementia have also changed over time.

Alzheimer's disease (AD) is the most common form of dementia, accounting for an estimated 60–80% of all dementia cases (11). Amyloid-β and tau are the two major pathological proteins that accumulate in the brains of patients with AD. These changes are accompanied by damage to brain tissue that ultimately results in cognitive impairment (12). In large-scale epidemiological studies conducted to date, obtaining an accurate estimate regarding the incidence and prevalence of AD was mainly hindered by the evolving clinical criteria for establishing these diagnoses (13) as well as by the relatively high dropout rates among cohort study participants with AD (14). Consequently, fewer studies have focused on evaluating the incidence and prevalence of AD specifically.

A community-based observational study conducted in Hisayama, Japan reported an increasing trend in the incidence and prevalence of AD (9), while a medical record-based study conducted in Rochester, NY showed a marginal declining trend in AD incidence (8). Studies enrolling a larger sample size as well as with methodological steps taken to lower drop-out rates are needed to understand trends in the incidence and prevalence of AD more comprehensively.

In this study, we aimed to evaluate annual trends in the incidence and prevalence of AD in Korea. We additionally investigated the optimal age cut-off values for establishing an effective diagnosis of AD as well as the effects of vascular risk factors with regard to the incidence and prevalence of AD using 10 years of diagnostic and prescription records obtained from the National Health Insurance System (NHIS) database representing the entire population of South Korea.

## Materials and Methods

### Participants

We conducted a retrospective nationwide population-based cohort study using data from a national health insurance claims database established by the National Health Insurance Service (NHIS) of Korea. The NHIS is a mandatory health insurance system in the Republic of Korea, which consists of National Health Insurance (NHI) recipients (encompassing 97% [52 million] of the Korean population), and Medical Aid (MA) recipients covering the remaining 3% of the population with low income (as of 2015). Since the adoption of the fee-for-service model for compensating healthcare providers by the NHIS, relevant information such as diagnostic codes, details of medical bills, prescription records, procedures, and demographic records have been compiled by the database administrators. The Health Insurance Review Agency provides quality control, evaluates healthcare performance, and reviews medical data (15).

This database is provided for research purposes by the “Big Data Steering Department” of the NHIS and encompasses information from the entire Korean population. Additional details regarding this database have been described previously (16). In this study, we evaluated the prevalence and incidence of AD within the NIHS database population aged 40 or older between January 2006 and December 2015.

This study was approved by the NHIS inquiry commission and received institutional review board (IRB) approval from the ethics review board at Gangnam Severance Hospital (IRB-No 3-2016-0307). The requirement for informed consent was formally waived for this secondary analysis in accordance with research and medical ethics conventions. The privacy of each participant was protected for the purpose of this analysis by anonymizing and deidentifying the evaluated national insurance claims data. All research was performed in accordance with relevant institutional, national, and international guidelines and regulations and was conducted in accordance with the principles of the Declaration of Helsinki and its later amendments.

### Definition

Individuals were diagnosed with AD based on the tenth revision codes of the International Statistical Classification of Diseases (ICD) for Alzheimer's dementia (F00, and G30) along with patients' prescription records for anti-dementia medication. Eligible medications were limited to acetylcholinesterase inhibitors (rivastigmine, galantamine, donepezil) or N-methyl-D-aspartate (NMDA)-receptor antagonist (memantine). The operational definition of AD was referenced in previous studies (17, 18).

Patients were diagnosed with hypertension when anti-hypertensive medications were prescribed as well as when the following ICD-10 codes were recorded: I10 (essential hypertension), I13 (hypertensive heart and renal disease), and/or I15 (secondary hypertension). Patients were defined as having type 2 diabetes if anti-diabetic drugs (insulin, sulfonylureas, metformin, meglitinides, thiazolidinediones, dipeptidyl peptidase-4 inhibitors, α-glucosidase inhibitors) were prescribed as well as in the presence of the ICD-10 following codes: E11 (non-insulin-dependent diabetes mellitus), E12 (malnutrition-related diabetes mellitus), E13 (other specified diabetes mellitus), or E14 (unspecified diabetes mellitus). Patients were defined as having dyslipidemia in the presence of ICD-10 code E78 (disorders of lipoprotein metabolism and other lipidemias).

### Statistical Analysis

All prevalence and incidence rates are expressed as the number of prevalent or incident cases per 1,000 persons along with associated 95% confidence intervals (CI). Age and sex were standardized using matched Korean Census data for the year 2010. The age cut-off value for the diagnosis of AD was defined as the point with the highest Youden's index (sensitivity + specificity – 1). Statistical analyses were performed using SAS statistical software (version 9.2, SAS Institute, Cary, NC).

## Results

### Incidence and Prevalence of AD

The Korean population aged 40 or older has increased gradually, from 19,732,425 in 2006 to 26,219,849 in 2015. The age-standardized incidence of AD during this timeframe has increased from 1.83 per 1,000 persons in 2006 to 5.21 per 1,000 in 2015. The age-standardized prevalence of AD increased from 3.17 to 15.75 per 1,000 persons between 2006 and 2015, respectively (Table 1). Both the incidence and prevalence of AD showed an annual increasing trend during this timeframe (*p* for trend <0.001). The highest annual increase in both incidence and prevalence occurred in 2009. The annual increase in the incidence and prevalence rates with regard to AD tended to be lower during the later study period (e.g., 2014, 2015) (Table 1).

**Table 1:** Age-standardized annual prevalence and incidence of Alzheimer's disease.

**Year**	**2006**	**2007**	**2008**	**2009**	**2010**	**2011**	**2012**	**2013**	**2014**	**2015**
***N***	19,732,425	20,331,062	21,264,533	22,016,750	22,753,047	23,463,877	24,216,010	24,878,739	25,557,674	26,219,849
Incidence (*N*)	30,439	37,398	48,067	67,965	72,455	81,560	94,701	105,978	111,542	122,580
Incidence rate (95% CI)	1.83 (1.81–1.86)	2.16 (2.14–2.18)	2.64 (2.61–2.66)	3.57 (3.54–3.6)	3.66 (3.64–3.69)	3.99 (3.96–4.02)	4.49 (4.46–4.52)	4.81 (4.78–4.84)	4.91 (4.88–4.93)	5.21 (5.18–5.24)
Increase from previous year (%)	N/A	36.8	28.3	35.6	19.8	18.3	19.1	19.4	13.2	13.7
Prevalence (*N*)	53,156	74,881	100,321	140,993	174,525	212,824	263,515	322,225	373,076	436,366
Prevalence rate (95% CI)	3.17 (3.14–3.19)	4.24 (4.21–4.27)	5.33 (5.3–5.36)	7.09 (7.06–7.13)	8.29 (8.25–8.33)	9.60 (9.56–9.64)	11.28 (11.24–11.33)	12.93 (12.89–12.98)	14.22 (14.17–14.26)	15.75 (15.7–15.8)
Increase from previous year (%)	N/A	18.0	22.2	35.2	2.52	9.0	12.5	7.1	2.1	6.1

*Incidence rate and prevalence rate are shown in event per 1,000 persons*.

*CI, confidence interval; NA, Not applicable*.

### Incidence and Prevalence of AD by Age

**Figure 1:**
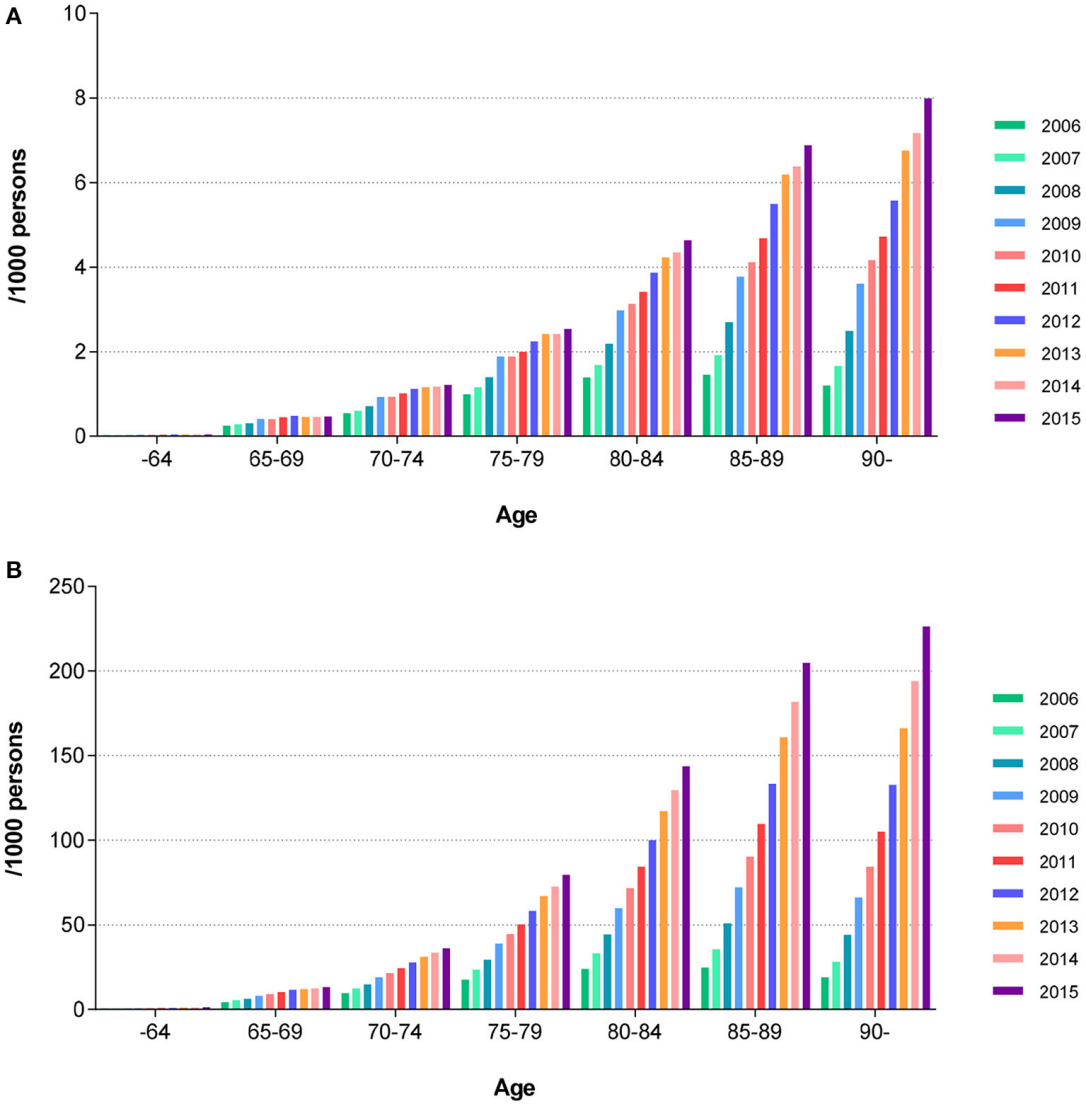
Sex standardized age-specific annual trends in the prevalence and incidence of AD. **(A)** Incidence of AD. **(B)** Prevalence of AD. AD, Alzheimer's disease.

The age-specific, sex-standardized incidence of AD showed an annual increase from 2006 to 2015 in all age groups. In the 65–69-year age group, the incidence of AD increased from 0.25 to 0.41 per 1,000 persons between 2006 and 2010, respectively, and further increased to 0.46 per 1,000 persons in 2015. In the 75–79-year age group, the incidence of AD increased from 1.00 to 1.88 per 1,000 persons between 2006 and 2010, respectively. A further increase to 2.54 per 1,000 persons was noted in 2015. In the 85–89-year age group, AD incidence increased from 1.46 to 4.12 per 1,000 persons between 2006 and 2010, respectively. A further increase to 6.88 per 1,000 persons was seen in 2015. The annual increase in the incidence rate of AD was higher in older age groups and during the early study period (from 2006 to 2009) (Figure 1A).

The age-specific, sex-standardized prevalence of AD showed a similar pattern as compared with the incidence patterns noted above. In the 65–69-year age group, the prevalence of AD increased from 4.25 to 9.04 per 1,000 persons between 2006 and 2010, respectively. A further increase to 13.2 per 1,000 persons was observed in 2015. In the 75–79-year age group, the prevalence of AD increased from 17.65 to 44.32 per 1,000 persons between 2006 and 2010, respectively. An increase to 79.49 per 1,000 persons was noted in 2015. In the 85–89-year age group, the prevalence of AD increased from 24.69 to 90.26 per 1,000 persons between 2006 and 2010, respectively. The prevalence further increased to 204.97 per 1,000 persons as of 2015. We note that the prevalence of AD increased in all evaluated age groups during the course of the study period. The annual increase in the prevalence rate of AD was higher in older age groups and during the early study period (from 2006 to 2009) (Figure 1B).

Based on the highest Youden's index value (sensitivity + specificity – 1), the cut-off value for the diagnosis of AD was 69 years with an area under the curve (AUC) of 0.92. The sensitivity, specificity, and Youden's index values for AD with respect to specific age groups are shown in Table 2.

**Table 2:** Sensitivity and specificity in predicting AD by the different age cut-off.

**AGE**	**Sensitivity**	**Specificity**	**^†^Youden index**
60	0.969	0.648	1.616
61	0.963	0.676	1.639
62	0.956	0.700	1.657
63	0.950	0.721	1.671
64	0.941	0.744	1.685
65	0.934	0.761	1.695
66	0.924	0.779	1.703
67	0.912	0.796	1.708
68	0.897	0.814	1.711
69	0.879	0.831	1.711^*^
70	0.863	0.845	1.708
71	0.845	0.857	1.702
72	0.822	0.870	1.692
73	0.794	0.883	1.677
74	0.754	0.899	1.652
75	0.713	0.911	1.625
Optimal cut-off	69		
AUC	0.922		

* *Designates the highest value of Youden index*.

*AD, Alzheimer's disease; AUC, area under the curve*.

† *Youden index = [Sensitivity + Specificity – 1]*.

### Incidence and Prevalence of AD in Subgroups With Vascular Risks

**Figure 2:**
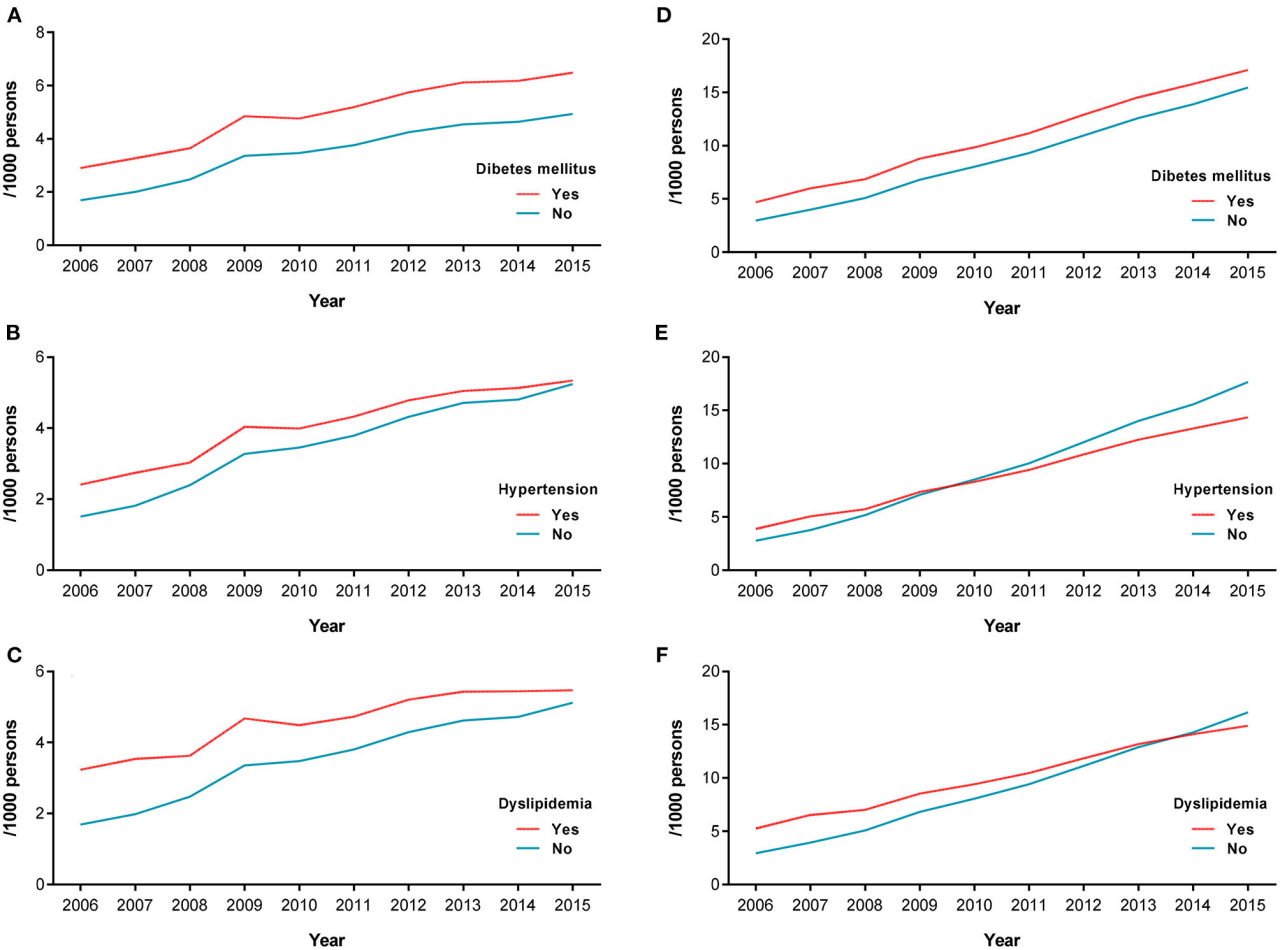
Annual incidence rate of AD in vascular disease subgroups. Incidence **(A)** and prevalence **(D)** of AD in the diabetes mellitus subgroup. Incidence **(B)** and prevalence **(E)** of AD in the hypertension subgroup. Incidence **(C)** and prevalence **(F)** of AD in the dyslipidemia subgroup. AD, Alzheimer's disease.

The annual incidence rates for AD in subgroups with hypertension, diabetes mellitus, and dyslipidemia were higher than in those without any vascular risks (Figure 2). For example, the age-standardized incidence rate for AD in subgroups with and without diabetes mellitus increased from 2.90 and 1.69 per 1,000 persons, respectively, in 2006 to 6.48 and 4.93 per 1,000 persons, respectively, in 2015. The gap between subgroups with and without diabetes showed a parallel trend during the course of the study period (Figure 2A).

In contrast, the gap between subgroups with and without hypertension as well as between those with and without dyslipidemia decreased over time during the course of the study period (Figures 2B,C). The age-standardized incidence rates for AD in subgroups with and without hypertension increased from 2.41 and 1.51 per 1,000 persons, respectively, in 2006 to 5.34 and 5.24, respectively, in 2015. Similarly, the age-standardized incidence rate of AD in subgroups with and without dyslipidemia showed an increase from 3.23 and 1.69, respectively, in 2006 to 5.47 and 5.12, respectively, in 2015.

The prevalence rate of AD in the subgroup with diabetes mellitus was higher than in those without diabetes mellitus during the study period, showing a stable gap difference (Figure 2D). The annual prevalence rate of AD in subgroups with hypertension and dyslipidemia were initially higher than in those without. However, the prevalence rate of AD in the subgroup with hypertension was conversely lower than in those without hypertension from 2010 onwards. The prevalence rate of AD in the subgroup with dyslipidemia was also lower than in those without dyslipidemia from 2014 onwards (Figures 2E,F).

## Discussion

In this nationwide cohort study conducted within the NHIS database representing the entire Korean population aged 40 years or older, we found increasing trends in the incidence and prevalence of AD, with older age groups showing higher incidence and prevalence rates and trends as compared with younger age at risk groups. In addition, the incidence of AD was higher in those with underlying vascular risks. Conversely, the prevalence of AD was found to be lower in the subpopulation with underlying hypertension and dyslipidemia.

The annual incidence and prevalence of AD increased throughout the study follow-up period. The aging of population may affect this trend. the proportion of those aged 65 or order in South Korea was 9.3% in 2006, and increased to 12.8% in 2015 (19). Considering the expected proportion of old aged population to be 40.4% in 2050 in South Korea (19), increasing trend in the prevalence and incidence of AD seems inevitable. In addition, increase in diabetes (20), obesity (21), and sedentary lifestyles (22) may also affect the increasing trend in the prevalence and incidence of AD. We noted a marked increase occurring during the early study period (i.e., 2009). This trend was also observed with respect to age-specific annual trends and in the vascular risk subgroup analysis. In 2008, the government of Korea announced the inception of the first national dementia plan, which included establishing dementia counseling centers at all community health centers. An early detection program as well as medical expense support services were initiated to encourage the early diagnosis of dementia. Consequently, 45.7% of the elderly population participated in this program (1). These national policies and efforts aimed at diagnosing dementia may have influenced the higher increase in the incidence and prevalence of AD that was seen during the course of the follow-up period.

The increasing trend seen in the oldest-age group is an important finding that is in accordance with the results of previous community-based studies (23, 24). This oldest group comprises the fastest growing segment of the population and the effects of vascular risk factors as well as a range of pathological characteristics are unique to this group. This warrants distinct prevention and management plans geared toward this age demographic (25).

Although the observed increases in incidence and prevalence rates were lowest in those aged <65 years, this age group showed a steady increase in incidence and prevalence rates, such that the incidence and prevalence rates of AD in this group were 1.8 and 3 fold higher, respectively, in 2015 as compared with 2006. Since early-onset AD is more likely to be caused by genetic factors (26), this finding may be attributed to a nationwide increase in the clinical diagnosis of early AD, which may have been misdiagnosed as a range of psychiatric disorders in the past (27).

The early detection and diagnosis of AD offers several benefits in terms of helping patients and their families, including opportunities to modify exposures to common risk factors as well as to make financial and legal arrangements before the occurrence of a significant decline in cognition (28). To enhance the early diagnosis of AD on a population level, many countries have established screening and diagnostic programs geared toward the entire older population (29). The Korean government has also executed a widely distributed diagnostic program composed of neuropsychological tests and clinical assessments to aid in the early diagnosis of dementia in the population aged 60 years or older; this program is administered through community-based dementia counseling centers (30). The optimal cut-off age for diagnosing AD using the NHIS database based on findings from within the entire Korean population enrolled in this study should be considered carefully so as to maximize the impact of limited social resources.

Interestingly, the lower prevalence of AD in the subpopulation with underlying hypertension or dyslipidemia (as compared to those without vascular risks) that has been observed in recent years was also noted in this study. This finding might have been influenced by the higher mortality rates seen in the subgroups with hypertension and dyslipidemia (31). In addition, antihypertensive and antidyslipidemic medications would lead to beneficial effects on the pathological process of AD (32–34). We also note that the awareness of hypertension and dyslipidemia had grown on a societal level during the course of the study period (35), while awareness surrounding diabetes has been reported to be relatively stable in South Korea (i.e., with room for improvement) (36, 37). The effect of more proactive diagnoses as well as the effective management of vascular risks on a population level may have played a role in the decreased prevalence of AD seen during the study period.

This study has some limitations. First, the diagnosis of AD was defined solely based on diagnostic codes and prescription history. Thus, there could be possible discrepancies the real-world situation and the information recorded in the claims database. For example, compared to the previous community-based epidemiologic studies in South Korea (38–40), the incidence and prevalence of AD tended to be lower in the current study. In addition to difference in statistical methodology, follow up duration, and different sample sizes, low medical utilization rate (~52.8%) of patients with dementia in South Korea may also contribute to the different outcomes (41). Second, a diagnosis of mixed dementia, which accounts for a significant portion of cases showing multifactorial features, was not accessed in the current study. This is an inevitable limitation in research efforts using large databases with diagnostic codes. The major strength of this study was the use of a real-world sample representing the entire national population; we evaluated over 2.5 million people aged 40 years or older. Compared to a sampled database, data derived from the entire population is more efficient at minimizing selection bias, thereby reflecting real-world situations more accurately and effectively.

Our findings indicate that the incidence and prevalence of AD is still growing in Korea. This trend is affected by the rapid aging of the population as well as by recent national policies for obtaining and promoting early diagnoses. More efforts aimed at population screening using this unique database resource as well as efforts aimed at modifying the underlying risks are warranted to reduce the burden of AD. Our findings thus guide future research directions and, if confirmed, will directly inform health policy decision-making.

## Data Availability Statement

The original contributions presented in the study are included in the article/supplementary material, further inquiries can be directed to the corresponding authors.

## Ethics Statement

The studies involving human participants were reviewed and approved by Institutional Review Board at Gangnam Severance Hospital. Written informed consent for participation was not required for this study in accordance with the national legislation and the institutional requirements.

## Author Contributions

MB contributed to conception and design, collection and assembly of data, data analysis and interpretation, and manuscript writing. H-KK contributed to data analysis and interpretation. KH contributed to conception and design, collection and assembly of data, and data analysis and interpretation. H-SK contributed to collection and assembly of data and data analysis and interpretation. HN contributed to collection and assembly of data and interpretation. CL contributed to conception and design, administrative support, and data analysis and interpretation. HC contributed to conception and design, administrative support, collection and assembly of data, data analysis and interpretation, and final approval of manuscript. All authors contributed to the article and approved the submitted version.

## Funding

This research was supported by a grant from a faculty research grant of Yonsei University College of Medicine for (6-2018-0068), Basic Science Research Program through the National Research Foundation (NRF) funded by the Ministry of Education (NRF2020R1F1A1076154, NRF2018R1D1A1B07049386, and NRF2022R1C1C1012535), and a grant of the Korea Health Technology R&D Project through the Korea Health Industry Development Institute (KHIDI) funded by the Ministry of Health & Welfare, Republic of Korea (Grant number: HI18C1159, HU20C0164).

## Conflict of Interest

The authors declare that the research was conducted in the absence of any commercial or financial relationships that could be construed as a potential conflict of interest.

## Publisher's Note

All claims expressed in this article are solely those of the authors and do not necessarily represent those of their affiliated organizations, or those of the publisher, the editors and the reviewers. Any product that may be evaluated in this article, or claim that may be made by its manufacturer, is not guaranteed or endorsed by the publisher.
